# Factors Influencing the Selection of a Physician for Dermatological Consultation in Saudi Arabia: A National Survey

**DOI:** 10.3390/healthcare13040404

**Published:** 2025-02-13

**Authors:** Fawwaz Freih Alshammarie, Abdullah Aziz Alenazi, Yasmin Saleh Alhamazani, Lenah Hassan Almarzouk, Mohammad Abdulkarim Alduheim, Duaa Abdullah Alahmadi, Kholah Fares Alshammari, Wael Saleh Alanazi

**Affiliations:** 1 Department of Dermatology, College of Medicine, University of Hail, Hail 55476, Saudi Arabia; 2 Security Forces Hospital Program, Riyadh 11481, Saudi Arabia; 3 Department of Dermatology, King Abdulaziz Medical City for National Guard, Riyadh 11426, Saudi Arabia; 4 Eastern Health Cluster, Dammam Medical Complex, Dammam 32253, Saudi Arabia; 5 King Faisal Specialist Hospital and Research Center, Riyadh 11211, Saudi Arabia; 6 King Fahd University Hospital, Khobar 34445, Saudi Arabia; 7 Hail Health Cluster, Hail 55471, Saudi Arabia

**Keywords:** dermatological, consultation, choosing dermatologist, factors

## Abstract

**Background:** Dermatology in healthcare is changed by technological advancements, patient awareness, and developing healthcare needs. In Saudi Arabia, choosing a dermatologist is influenced by various factors, including patient satisfaction, digital technology use, and the physician’s characteristics and qualities. **Purpose:** The major aim of this research was to evaluate factors influencing patients’ choice of dermatologists in Saudi Arabia, including past experiences, reputation, social media influence, and access to care. **Methods:** A comprehensive national survey was conducted in Saudi Arabia. The research included 1038 participants using stratified sampling, predominantly from the 18–25 age group (67.1%). The distribution covered all regions of Saudi Arabia, with a larger portion from the Central and Western regions. The survey included quantitative and qualitative questions, assessing factors such as previous experiences with dermatologists, the importance of physician reputation, the role of digital platforms, and access to care. The data were analyzed using the Statistical Package for Social Studies (SPSS 22; IBM Corp., New York, NY, USA), with continuous variables expressed as mean ± standard deviation and categorical variables as percentages. **Results:** Our study highlighted a strong preference for dermatologists based on previous positive experiences (95.3%) and reputable dermatologists (95.2%). Factors like social media presence were less influential. Demographics matter, with younger and female participants favoring female doctors. The education level affects preference factors like ads, and the convenience of their location holds minimal sway. Recommendations from peers and healthcare professionals carry significant weight in the decision. **Conclusions**: Positive patient experiences and professional reputation suggest that these are the factors that affect patients’ selection of a dermatologist the most. The research findings indicated that while digital platforms are important for information dissemination, they play a minimal role in the actual selection process. The findings can guide dermatologists and healthcare policymakers in enhancing patient-centered care and adapting to evolving patient preferences in the digital age.

## 1. Introduction

The global occurrence of dermatological diseases has emphasized the need for specialized care. Skin diseases are the fourth leading contributor to the worldwide burden of nonfatal diseases, as highlighted by studies such as those by Hay et al. and Karimkhani et al. (2017) [1]. These situations have an effect on approximately 84.5 million individuals within the US alone, underscoring the important role of dermatologists in addressing those challenges [2]. Dermatologists play a pivotal function in quantifying the weight of pores and skin ailments and advocating for effective remedies, leading to improved patient care and international fitness initiatives.

Patient satisfaction is an essential determinant of care quality, influencing treatment adherence and scientific effects. However, the role of patient satisfaction in selecting a dermatologist remains underexplored. While preceding research like that by Parraga and Feldman (2024) [3] has addressed patient opinions and pride, there is a need to specifically investigate how those factors affect the choice-making system when deciding on a dermatologist.

Recent advancements in telemedicine have redefined patient preferences in selecting healthcare providers, with convenience and shorter wait times influencing expectations [4]. Furthermore, social media platforms increasingly shape patient–dermatologist interactions, influencing choices and fostering new dynamics in healthcare [3]. However, previous studies, including that by Berendsen et al. (2009) [5], lack specificity to dermatological contexts and require updating to align more with these. Access to dermatology appointments remains a large determinant, with elements like the area, patient complaints, clinician type, and insurance/payment method playing a position [6]. Equitable admission to dermatology offerings is particularly important for patients with restricted resources. 

A cross-sectional survey revealed that patients tend to prioritize professionally relevant factors, which include qualifications, enthusiasm, and a reputation for delivering remarkable care, whereas the non-professional characteristics of the dermatologist play a much smaller role in their decision-making. Despite these insights, gaps remain in understanding how various factors, including social media, telemedicine, and patient satisfaction, interact to shape patient decisions.

The present study seeks to discover those various factors and their interactions, with the aid of distributing questionnaires to participants. This approach aims to improve access to specialized dermatological care through identifying the important determinants of dermatologist selection, thereby optimizing the clinical effects and increasing superb care delivery. By addressing these essential components, this study contributes to the developing body of literature on dermatological healthcare admission and patient decision-making.

## 2. Materials and Methods

### 2.1. Study Design and Participants

A comprehensive national survey was conducted for this study, targeting 1038 participants through stratified sampling. The majority of participants were aged 18–25 years (67.1%), with a higher proportion of females (76.5%). Most participants were single (77.8%), held a bachelor’s degree (62.6%), and were students (50.7%). The survey covered all regions of Saudi Arabia, with a larger proportion from the Central and Western regions. Additionally, a significant majority (68.7%) did not have medical insurance. Analyzing the data by age and gender enhances the accuracy and inclusivity of dermatology research, ensuring the findings are applicable to diverse groups. This approach also advances understanding, diagnosis, and treatment of skin conditions across different populations.

### 2.2. Survey Method

The survey employed a structured questionnaire designed to capture both quantitative and qualitative data. This questionnaire was developed following a comprehensive literature review and consultations with experts to ensure that all relevant factors influencing the choice of dermatologist were addressed. It included demographic information such as age, gender, marital status, education level, employment status, region of residence, and medical insurance status. Additionally, it covered factors like previous experiences with dermatologists, the dermatologist’s reputation, the influence of social media, accessibility, and recommendations from peers and healthcare professionals. Patient preferences regarding specific dermatologist characteristics such as gender, board certification, years of experience, and affiliation with reputable hospitals were also included. The questionnaire is provided in the Supplementary Materials.

### 2.3. Study Period and Data Collection

Data collection was conducted over three months, from January to March 2023. Participants were approached through multiple channels, including online platforms, social media, and in-person at public locations such as malls and universities. Informed consent was obtained from all participants before they completed the survey [7,8,9,10,11,12,13,14].

### 2.4. Statistical Analysis

Data were analyzed using Statistical Package for Social Studies (SPSS 22; IBM Corp., New York, NY, USA). Continuous variables were expressed as mean ± standard deviation, and categorical variables were expressed as percentages. A five-point Likert scale was applied to these questions to rate a respondent’s preference (strongly disagree = 1, disagree = 2, neutral = 3, agree = 4, strongly agree = 5). The Mann–Whitney test was used for continuous variables without a normal distribution. The Shapiro–Wilk test was used to assess the normal distribution of the variables. A *p*-value < 0.05 was considered statistically significant.

## 3. Results

### 3.1. Demographic Characteristics

**Figure 1:**
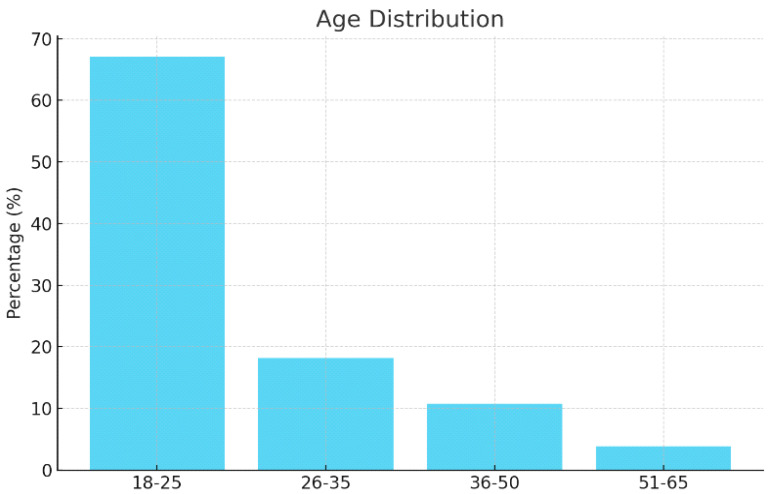
Demographic characteristics—age distribution.

Table 1 shows the demographic characteristics of the participants. A total of 1038 participants from all regions of Saudi Arabia were included in this study. More than two-thirds of the participants (67.1%) were in the age group of 18–25 years, and 76.59% were women. The majority of the participants (77.8%) were single. As for the educational attainment, almost two-thirds of the participants (62.6%) had bachelor’s degrees, more than half were students (50.7%), and the rest were employed at the time of the survey (29.2%). Moreover, 32.8% of the participants had children. According to the residence region, more than half of the participants lived in the Central and Western regions. More than two-thirds of the participants did not have medical insurance. Refer to Figure 1 for a graphical representation of these characteristics.

### 3.2. Factors Influencing Dermatologist Selection

Clarifications have been made in the figure legends to address differences such as “based on friend’s advice” and “through a friend’s recommendation”. Refer to Table 4 for education-level comparisons.

**Figure 2:**
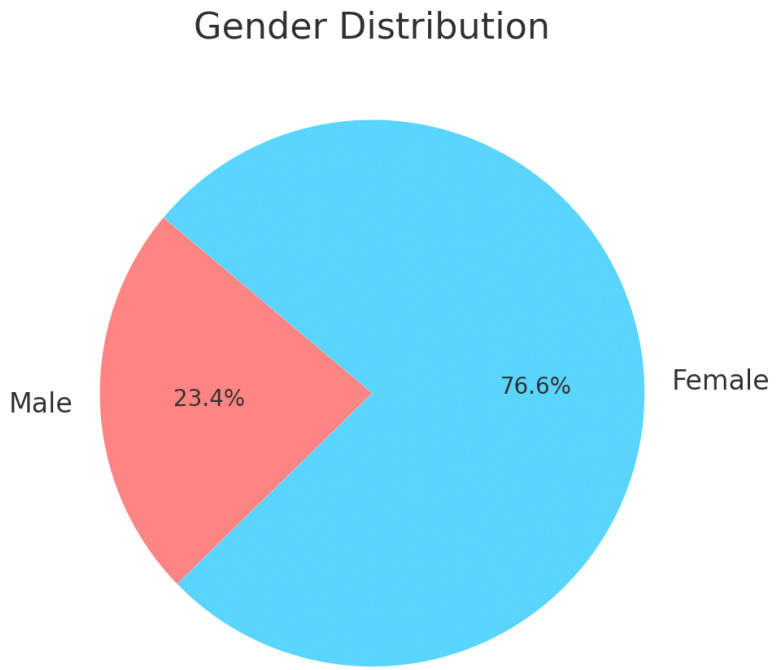
Demographic characteristics—gender distribution.

**Figure 3:**
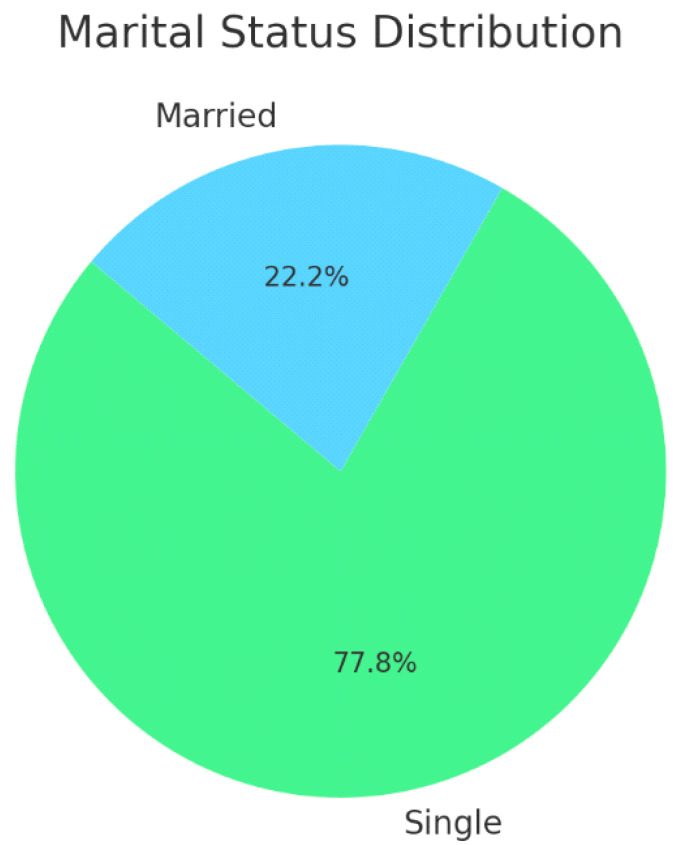
Demographic characteristics—marital status distribution.

**Figure 4:**
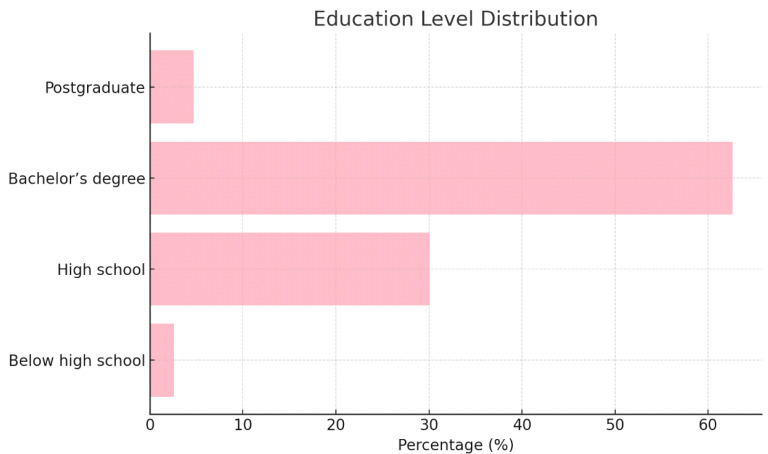
Demographic characteristics—education level distribution.

Figure 2, Figure 3 and Figure 4 illustrate the gender distribution, marital status and education levels of the population, respectively.

**Figure 5:**
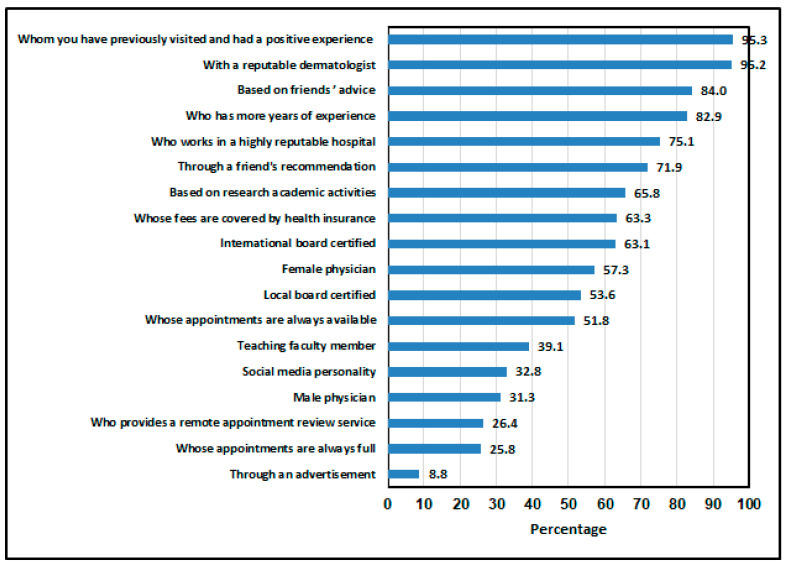
The most important factor for selecting a dermatologist.

Figure 5 illustrates the most significant factors influencing the selection of a dermatologist. It can be observed that the most influential factor was having previously visited a dermatologist and having had a positive experience (95.3%), closely followed by the reputation of the dermatologist (95.2%). In contrast, advertisements were the least influential factor, affecting only 8.8% of the respondents’ decisions.

**Figure 6:**
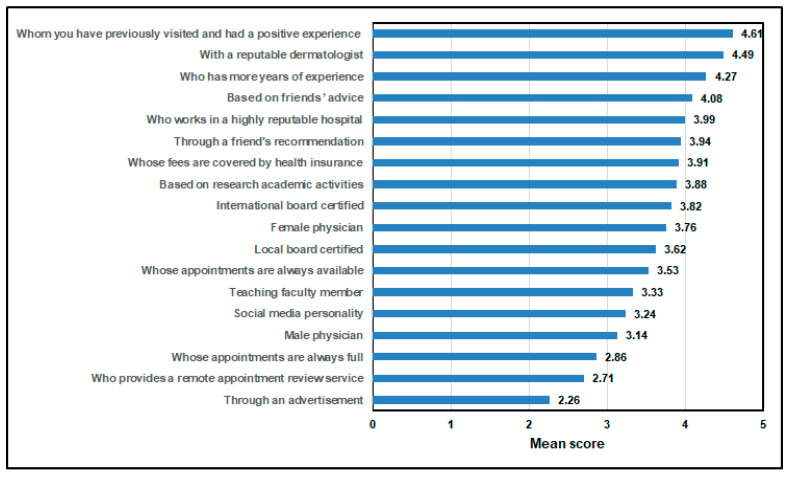
Most preferred factors affecting choice of dermatologist [response ranges from 1 (strongly disagree) to 5 (strongly agree)].

Figure 6 shows the mean score of the factors affecting the preferred dermatologist. The factors of having visited a dermatologist previously and whether the experience was positive had the highest mean scores, with a mean score of 4.6 and 4.4, respectively. In contrast, the factor of individuals being reached through advertisements had the lowest mean score (2.2).

Table 2 highlights the differences in the dermatologist preferences across the age groups. A significant difference in the dermatologist preferences is observed between individuals aged 25 years and older compared to those under 25 years. These differences are notable in factors such as friends’ advice (*p* = 0.041), local board certification (*p* = 0.028), social media personality (*p* = 0.006), preference for male physicians (*p* < 0.001), preference for female physicians (*p* < 0.001), working in a highly reputable hospital (*p* < 0.001), and having previously visited and had a positive experience with a dermatologist (*p* < 0.001).

Table 3 shows the differences in the dermatologist preferences according to gender. There is a significant difference in the dermatologist preferences between the males and females based on several factors, including social media personality, male physician, female physician, a physician working in a highly reputable hospital, and a physician previously visited with a positive experience. The corresponding *p*-values for these factors are <0.001, <0.001, <0.001, 0.016, and <0.001, respectively.

Table 4 highlights the differences in the dermatologist preferences based on the level of education. A significant variation is observed between the individuals with a high school education or below and those with a bachelor’s degree or higher. This difference is evident in the preferences for local board-certified male physicians, female physicians, research academic activities, and physicians working in highly reputable hospitals, with *p*-values of 0.001, 0.001, <0.001, 0.004, and 0.046, respectively.

## 4. Discussion

The study outcome revealed that patients in Saudi Arabia choose dermatologists based on positive experiences (95.3%) and reputation (95.2%). This aligns with Burroway et al.’s (2019) findings, which emphasize reputation and experience over demographics. While demographics play a smaller role, age and gender influence preferences, with younger individuals and females preferring female physicians and social media personalities [7]. Patients prioritize factors like language, medical school, and online reviews over age, gender, and race. Personal experience and word-of-mouth recommendations are more influential than digital platforms in choosing a dermatologist [8-9]. While Geist et al. found 95.2% of patients on social media seek dermatology-related information, Albeshri et al. [9] showed only 21% choose a dermatologist through social media, with 66% preferring familiar doctors. This suggests that while social media has an influence, many patients still prefer dermatologists they are already familiar with through other means.

Through a retrospective analysis, Tripathi et al.’s analysis (2007–2015) revealed disparities in US outpatient dermatology services, with Hispanic and black patients, males, and those in the Midwest receiving less care. Higher levels of education and income were linked to increased access to care, highlighting demographic and socioeconomic influences [10,11,12]. Medicaid enrollees were less likely to receive skin condition diagnoses, especially from dermatologists, compared to those with private coverage, highlighting the need to address access disparities. Disparities in dermatology care exist, with factors like insurance, age, gender, race, and geography influencing access. Research byTripathi (2018) highlighted these disparities, with minorities predicted to comprise the majority of the US population by 2044. These studies emphasize the need for addressing access barriers and promoting equitable care [10].

Patient satisfaction plays a critical role in dermatology care, with a direct impact on clinical outcomes, patient retention, and the likelihood of medical malpractice claims. Highly satisfied patients generally experience better outcomes, particularly in managing chronic skin conditions, as they are more likely to adhere to treatment plans. Conversely, dissatisfied patients are more prone to seek care elsewhere. Research by Al Shammrie (2022) underscores the importance of maximizing patient satisfaction, noting its inverse correlation with medical malpractice claims. By prioritizing patient satisfaction, dermatologists can not only enhance the quality of care but also mitigate legal risks [13]. A study by Poulos (2022) used the Dr Score survey to assess the patient satisfaction in dermatology. An analysis of 394 office visits revealed high satisfaction in aspects like timely test results (82%), patient inclusion in decision-making (76%), clear instructions (75%), and effective communication (75%). The areas for improvement included follow-up on problems (22%), treatment success (20%), and time spent with patients (20%). Overall, 53% of the patients gave a perfect score, and 76% scored 8 or higher, emphasizing the importance of communication and time spent with patients in quality dermatological care [11].

To improve patient satisfaction and attract a larger patient base, dermatologists can strengthen their professional reputation by pursuing continuous education and training. Implementing systems to track the quality of care and patient feedback is essential for ensuring consistent improvements. Focusing on patient-centered communication and fostering trust is crucial, as is establishing robust patient feedback systems and developing personalized care plans. Additionally, utilizing digital tools for convenient appointment scheduling and management can significantly enhance the patient experience. Policymakers and dermatologists can work together to address ethnic disparities in healthcare by implementing policies to ensure equitable access to dermatological care, expanding insurance coverage, simplifying the insurance claim process, and promoting wider access to care in underserved areas. According to Katta et al. (2023), maintaining standards in patient care can be achieved through standardizing treatment protocols, consistent peer reviews, and clinical audits. Encouraging continuous professional development among dermatologists through training, workshops, and research support is crucial for advancing the field and enhancing patient care quality [14].

In Saudi Arabia, cultural and language barriers between patients and expatriate healthcare workers, including dermatologists, present substantial challenges to effective healthcare delivery. A significant portion of healthcare professionals in the country come from diverse cultural backgrounds and may lack a comprehensive understanding of the unique blend of Arabic traditions and Islamic influences that shape Saudi culture. This cultural knowledge gap can lead to cultural incompetence, culture shock, and communication difficulties, ultimately resulting in patient dissatisfaction and a decline in the quality of care provided. Furthermore, the language barrier between Arabic-speaking patients and English-speaking expatriate healthcare workers exacerbates the issue. To address these challenges, there is an urgent need for educational and orientation programs aimed at healthcare workers, focusing on Saudi culture and language. These programs should emphasize the importance of cultural understanding and effective communication within healthcare settings. By equipping healthcare professionals with the necessary cultural knowledge and skills, providers can deliver more effective, patient-centered, and culturally sensitive care, ultimately improving the overall quality of healthcare in Saudi Arabia.

## 5. Conclusions

It is essential to consider the ethical implications of social media use by healthcare professionals. While an active social media presence can enhance visibility and accessibility, it does not always equate to trustworthiness or competence. Dermatologists must navigate these platforms carefully, maintaining professionalism and ensuring that their promotional activities align with ethical standards. Establishing clear guidelines for social media use in healthcare is critical to balancing accessibility with ethical responsibility.

Research suggests that advertising has a limited effect on patient trust and decision-making when choosing a healthcare provider. In contrast, the healthcare professional’s reputation significantly influences patient preferences, often surpassing the importance of social media presence. To ensure accessibility while maintaining ethical standards, it is essential to establish clear guidelines for the use of social media in healthcare. The American Medical Association (AMA) recommends that doctors protect personal information through privacy settings and maintain clear boundaries between their personal and professional life. Additionally, healthcare professionals need to be aware of the ethical tensions that arise from blurring these lines. This is especially true regarding patient confidentiality and professional boundaries. In summary, while social media can act as a powerful tool for engagement and information dissemination, it also poses risks that must be carefully considered. By adhering to established ethical guidelines and promoting a culture of professionalism online, dermatologists can use social media effectively while maintaining the trust that patients have placed in them.

## Figures and Tables

**Table 1:** Demographic characteristics of the participants (n = 1038).

		Number	%
Age	18–25	697	67.1
	26–35	189	18.2
	36–50	112	10.7
	51–65	40	3.8
Gender	Male	243	23.4
	Female	795	76.5
Marital status	Single	808	77.8
	Married	230	22.1
Educational level	Below high school	27	2.6
	High school	312	30.0
	Bachelor’s degree	650	62.6
	Postgraduate	49	4.7
Occupational status	Student	527	50.7
	Employed	284	27.3
	Unemployed	158	15.2
	Other	69	6.6
Region of residence	Central	317	30.5
	Western	243	23.4
	Eastern	268	25.8
	Northern	122	11.7
	Southern	88	8.4
Do you have medical insurance?	Yes	325	31.3
	No	713	68.6

**Table 2:** Differences in factors affecting dermatologist preferences according to age group (n = 1038).

Dermatologist Preferences	Age ≥ 25 y		Age < 25 y		*p* Value
	Mean	SD	Mean	SD	
Found through an advertisement	2.23	0.92	2.33	0.97	0.176
A reputable dermatologist	4.51	0.61	4.46	0.68	0.327
Based on friends’ advice	4.11	0.72	4.02	0.72	0.041 *
Through a friend’s recommendation	3.93	0.87	3.95	0.89	0.533
Provides a remote appointment review service	2.68	1.17	2.77	1.08	0.192
Local board-certified	3.67	0.79	3.53	0.79	0.028 *
International board-certified	3.84	0.85	3.79	0.77	0.247
Has more years of experience	4.27	0.79	4.27	0.77	0.899
Social media personality	3.29	0.88	3.13	0.88	0.006 *
Male physician	3.02	0.96	3.38	0.92	<0.001 *
Female physician	3.95	0.93	3.36	0.91	<0.001 *
Based on research, academic activities	3.90	0.86	3.84	0.84	0.304
Teaching faculty member	3.36	0.93	3.28	0.89	0.249
Their appointments are always full	2.88	1.02	2.82	1.00	0.466
Their appointments are always available	3.53	0.92	3.52	0.88	0.866
Works in a highly reputable hospital	4.06	0.80	3.86	0.83	<0.001 *
Has been previously visited and a positive experience was had	4.69	0.56	4.43	0.72	<0.001 *
Their fees are covered by health insurance	3.94	0.93	3.86	0.88	0.149

* Significant *p*-value.

**Table 3:** Differences in dermatologist preferences according to Gender (n = 1038).

Dermatologist Preferences	Male		Female		*p* Value
	Mean	SD	Mean	SD	
Found through an advertisement	2.30	1.02	2.25	0.91	0.822
A reputable dermatologist	4.45	0.66	4.51	0.63	0.200
Based on friends’ advice	4.04	0.72	4.10	0.72	0.364
Through a friend’s recommendation	4.00	0.89	3.92	0.87	0.182
Provides a remote appointment review service	2.81	1.13	2.68	1.14	0.109
Local board-certified	3.60	0.82	3.63	0.78	0.790
International board-certified	3.88	0.81	3.80	0.83	0.148
Has more years of experience	4.27	0.80	4.27	0.78	0.949
Social media personality	3.06	0.89	3.29	0.87	<0.001 *
Male physician	3.69	0.87	2.97	0.93	<0.001 *
Female physician	3.06	0.89	3.97	0.88	<0.001 *
Based on research, academic activities	3.84	0.88	3.90	0.85	0.421
Teaching faculty member	3.34	0.92	3.33	0.91	0.795
Their appointments are always full	2.93	1.05	2.84	1.01	0.167
Their appointments are always available	3.47	0.93	3.55	0.90	0.449
Works in a highly reputable hospital	3.88	0.84	4.03	0.80	0.016 *
Has been previously visited and a positive experience was had	4.45	0.71	4.66	0.60	<0.001 *
Their fees are covered by health insurance	3.87	0.93	3.92	0.91	0.436

* Significant *p*-value.

**Table 4:** Differences in dermatologist preference according to level of education (n = 1038).

Dermatologist Preferences	High School or Below		Bachelor or Higher		*p* Value
	Mean	SD	Mean	SD	
Found through an advertisement	2.33	0.95	2.23	0.93	0.101
A reputable dermatologist	4.49	0.65	4.50	0.63	0.803
Based on friends’ advice	4.07	0.70	4.09	0.73	0.511
Through a friend’s recommendation	3.93	0.87	3.94	0.88	0.717
Provides a remote appointment review service	2.68	1.12	2.72	1.15	0.618
Local board-certified	3.54	0.79	3.66	0.79	0.001 *
International board-certified	3.80	0.82	3.83	0.83	0.365
Has more years of experience	4.29	0.81	4.26	0.77	0.312
Social media personality	3.22	0.89	3.25	0.88	0.962
Male physician	2.99	1.02	3.20	0.93	0.001 *
Female physician	3.96	0.91	3.66	0.98	<0.001 *
Based on research, academic activities	3.99	0.83	3.83	0.86	0.004 *
Teaching faculty member	3.40	0.90	3.30	0.92	0.154
Their appointments are always full	2.89	1.00	2.85	1.03	0.412
Their appointments are always available	3.50	0.92	3.54	0.90	0.318
Works in a highly reputable hospital	4.06	0.81	3.96	0.81	0.046 *
Has been previously visited and a positive experience was had	4.63	0.62	4.60	0.64	0.373
Their fees are covered by health insurance	3.87	0.96	3.93	0.90	0.449

* Significant *p*-value.

## Data Availability

The raw data supporting the conclusions of this article will be made available by the authors on request.

## Supplementary Materials

The following supporting information can be downloaded at https://www.mdpi.com/article/10.3390/healthcare13040404/s1.
