# Supplementary material for: Factors Influencing the Selection of a Physician for Dermatological Consultation in Saudi Arabia: A National Survey
Source: Healthcare (Basel). 2025 Feb 13;13(4):404. doi: 10.3390/healthcare13040404 (PMC11855030; doi:10.3390/healthcare13040404)
Supplement: Supplementary file 1 [file healthcare-13-00404-s001.zip › healthcare-3344356-supplementary.pdf]

Would you like to participate in the research?

- I want to participate
- I don't want to participate

Region :

- Northern Region
- Central Region
- Eastern Region
- Western Region
- Southern Region

Gender :

- Male
- Female

Age :

- Less than 20
- 20 - 30
- 31 - 40
- 41 - 50
- More than 50

Educational level :

- Elementary school
- Middle school
- High school
- Academic
- Postgraduate education

Have you ever visited a dermatology clinic?

- Yes
- No

Type of clinic :

- Governmental clinic
- Private clinic

Reason for visiting the clinic:

- cosmetic purpose
- Medical condition

Gender of the doctor:

- Female
- Male

How satisfied were you with the appointment time?

- Very satisfied
- Satisfied
- Unsatisfied
- Very unsatisfied
- Neutral

How satisfied were you with the waiting time for the consultation?

- Very satisfied
- Satisfied
- Unsatisfied
- Very unsatisfied
- Neutral

How satisfied were you with the explanation of your condition by the dermatologist?

- Very satisfied
- Satisfied
- Unsatisfied
- Very unsatisfied
- Neutral

How satisfied were you with the time spent by the dermatologist on the consultation?

- Very satisfied
- Satisfied
- Unsatisfied
- Very unsatisfied
- Neutral

How satisfied were you with the discussion of your condition including the information provided and treatment methods?

- Very satisfied
- Satisfied

- Unsatisfied
- Very unsatisfied
- Neutral

How satisfied were you with the dermatologist's response to your questions?

- Very satisfied
- Satisfied
- Unsatisfied
- Very unsatisfied
- Neutral

Did the dermatologist do a skin examination for you?

- Yes
- No

How satisfied were you with the skin examination?

- Very satisfied
- Satisfied
- Unsatisfied
- Very unsatisfied
- Neutral

Did the dermatologist take photos for medical purposes?

- Yes
- No

Was a skin biopsy taken?

- Yes
- No

Provider listens to me carefully, and respects my decisions and privacy?

- Strongly agree
- Agree
- Disagree
- Strongly disagree
- Neutral
